# *In silico* and Genetic Analyses of Cyclic Lipopeptide Synthetic Gene Clusters in *Pseudomonas* sp. 11K1

**DOI:** 10.3389/fmicb.2019.00544

**Published:** 2019-03-19

**Authors:** Hui Zhao, Yan-Ping Liu, Li-Qun Zhang

**Affiliations:** ^1^Department of Plant Pathology and MOA Key Laboratory of Pest Monitoring and Green Management, China Agricultural University, Beijing, China; ^2^National Laboratory of Biomacromolecules, CAS Center for Excellence in Biomacromolecules, Institute of Biophysics, Chinese Academy of Sciences, Beijing, China

**Keywords:** *Pseudomonas*, genome mining, secondary metabolites, cyclic lipopeptides, NRPS, rhizosphere, antifungal activity, biofilm formation

## Abstract

*Pseudomonas* sp. 11K1, originally isolated from rhizosphere, possesses inhibitory activity against plant pathogenic fungi and bacteria. Herein, the genome of strain 11K1 was sequenced and subjected to *in silico*, mutational, and functional analyses. The 11K1 genome is 6,704,877 bp in length, and genome mining identified three potential cyclic lipopeptide (CLP) biosynthetic clusters, subsequently named brasmycin, braspeptin, and brasamide. Insertional and deletion mutants displayed impaired brasmycin and braspeptin production, and lost antifungal activity, but retained antibacterial activity against *Xanthomonas oryzae*. The structures of these two active CLPs were predicted based on adenylation (A) domains. Brasmycin is composed of nine amino acids and belongs to the syringomycin class, while braspeptin is a 22 amino acid cyclic peptide belonging to the tolaasin group. Matrix-Assisted Laser Desorption/Ionization Time-of-Flight (MALDI-TOF) mass spectrometry analysis revealed that brasmycin and braspeptin have different molecular weights compared with known syringomycin and tolaasin members, respectively. Mutation of brasmycin and braspeptin gene clusters affected both biofilm formation and colony morphology. Collectively, these results indicate that *Pseudomonas* sp. 11K1 produces two novel CLPs that may help bacteria compete for nutrients and niches in the environment.

## Introduction

*Pseudomonas* spp. are ubiquitous in aquatic and terrestrial habitats, especially plant rhizospheres. Rhizosphere-derived pseudomonads have received much attention in recent decades because many are able to suppress plant diseases (Bender et al., 1999; Raaijmakers et al., 2002; Weller, 2007). Production of antimicrobial metabolites is one of the major mechanisms by which pseudomonads prevent microbial infection. *Pseudomonas* species exhibit extensive metabolic versatility and produce a remarkable spectrum of antimicrobial metabolites including cyclic lipopeptides (CLPs), 2,4-diacetylphloroglucinol (DAPG), phenazines (PHZs), pyrrolnitrin (PRN), pyoluteorin (PLT), 2,5-dialkylresorcinol, quinolones, gluconic acid, rhamnolipids, siderophores, and hydrogen cyanide (Gross and Loper, 2009; Masschelein et al., 2017).

Comparative genomics studies have revealed substantial diversity among different species of the *Pseudomonas* genus, and even among strains belonging to the same species (Loper et al., 2012). Many novel antimicrobial compounds have been discovered using genome analysis, including CLPs (de Bruijn et al., 2007; Gross and Loper, 2009; Nikolouli and Mossialos, 2012). CLPs are amphiphilic molecules composed of a cyclic oligopeptide lactone ring coupled to a fatty acid tail (Raaijmakers et al., 2010). CLPs are synthesized by non-ribosomal peptide synthases (NRPSs), large enzymes that are composed of a series of modules. Each module is a building block for the stepwise incorporation of an amino acid in the CLP peptide moiety and consists of an adenylation (A) domain, a thiolation (T) domain, and a condensation (C) domain. The A domain is responsible for amino acid selection and activation, the thiolation (T) domain catalyzes thioesterification of the activated amino acid, and C domain promotes peptide bond formation between two neighboring substrates to elongate the peptide chain. Based on their structural relationships, CLPs produced by *Pseudomonas* spp. are divided into at least six categories (Raaijmakers et al., 2006; Gross and Loper, 2009). Recently, Geudens and Martins (2018) further classified *Pseudomonas* spp. CLPs into 14 major groups, according to oligopeptide length and structure. Most CLPs produced by *Pseudomonas* species exhibit antifungal and antibacterial activity, and some possess antioomycete, antiviral, antiprotozoan, and antitumor activities (Masschelein et al., 2017).

Cyclic lipopeptides exert their antimicrobial activities by targeting either cell wall biosynthesis or cell membrane integrity. Friulimicin B from *Actinoplanes friuliensis* and the recently discovered CLP malacidin encoded in soil microbiomes inhibit cell wall biosynthesis (Schneider et al., 2009; Hover et al., 2018). Daptomycin produced by *Streptomyces roseosporus* and tridecaptin A1 produced by *Bacillus* and *Paenibacillus* species exert antimicrobial activity by disrupting membrane integrity (Straus and Hancock, 2006; Cochrane et al., 2016). Also *Pseudomonas* CLPs such as syringopeptins, cormycin, and massetolides exert their antibiosis activity by affecting membrane integrity. Overall, the molecular targets of most CLPs remain unknown (Masschelein et al., 2017).

In addition to antimicrobial activity, many CLPs produced by *Pseudomonas* spp. are involved in a wide range of other biological functions such as motility, biofilm formation, and virulence. Most CLPs produced by *Pseudomonas* spp. affect bacterial motility and biofilm formation as biosurfactants (Raaijmakers et al., 2010). Syringomycin and syringpeptin contribute to the virulence of *P. syringae* pv. *syringae* in cherry fruits (Scholz-Schroeder et al., 2001), and CLP sessilin hampers orfamide production in *Pseudomonas* sp. CMR12a (D’Aes et al., 2014).

We previously isolated *Pseudomonas* sp. 11K1 from *Vicia faba* rhizosphere in Yunnan province. This strain exhibits strong inhibitory activity against plant pathogens, including the fungal pathogen *Botryosphaeria dothidea* that causes canker of grapevines, and the bacterial pathogen *Xanthomonas oryzae* RS105 that causes bacterial blight of rice. In the present work, we sequenced the genome of strain 11K1, performed *in silico* and genetic analyses of putative secondary metabolite biosynthetic clusters, and identified two CLP clusters contributing to the antifungal activities displayed by this strain.

## Materials and Methods

### Strains and Growth Conditions

Bacterial strains and plasmids used in this study are listed in Supplementary Table S1. *Pseudomonas* sp. 11K1 and its mutants were grown in lysogenic broth (LB), AB medium (ABM), or amended King’s B medium (Proteose peptone No.3, 10 g; K_2_HPO_4_, 1.5 g; MgSO_4_ ⋅ 7H_2_O, 1.5 g; glucose, 20 g; KBG) at 28°C. *Escherichia coli* cells were grown at 37°C in LB medium. *B. dothidea* was cultured in PDA medium, and *X. oryzae* RS105 cells were grown at 28°C in LB or PDA medium. Where indicated, media was supplemented with ampicillin (50 μg/mL) or kanamycin (50 μg/mL) and/or 5-bromo-4-chloro-3-indolyl-D-galactopyranoside (X-Gal; 40 μg/mL).

### Genome Sequencing and Assembly

Genomic DNA from 11K1 was prepared from a culture grown from a single colony using a DNA purification kit (Promega, Beijing, China) and sequenced on a PacBio RS II platform (Pacific Biosciences, Menlo Park, CA, United States) following the manufacturer’s instructions. *De novo* assembly of PacBio reads was performed using the Smartanalysis pipeline v2.3.0 in conjunction with the HGAP assembly protocol, and additional assembly was performed with minimus2. Open reading frames were identified and annotated using GeneMarkS, rRNAs were predicted by Barrnap and RNAmmer, and tmRNAs and tRNAs were predicted using ARAGORN and tRNAscan-SE, respectively. Other non-coding RNAs were predicted and classified by infernal conjunction with Rfam. The functions of genes were also annotated using the COG, KEGG, Pfam, TIGRFAMs, and SwissProt databases.

### Genome Analysis of Putative Secondary Metabolite Clusters

The genome of the 11K1 strain was analyzed using the Antibiotics and Secondary Metabolite Analysis Shell (antiSMASH) pipeline (Blin et al., 2017). ClusterFinder and Use ClusterFinder algorithms were employed for biosynthetic gene cluster (BGC) border prediction and analysis. Complete genome sequences of 11K1, four strains of *P. brassicacearum*, and 15 well-studied biocontrol agents belonging to other species of the *Pseudomonas* genus were also analyzed using Extra Features (KnownClusterBlast, SubClusterBlast, and ActiveSiteFinder).

### Mutation of Putative Secondary Metabolite Gene Clusters

Fragments of genes to be inactivated were PCR-amplified using primer pairs and PCR conditions listed in Supplementary Table S2. PCR products were cloned into the p2P24-Km suicide vector (Yan et al., 2017) to generate insertional mutant constructs, designated as p2P24-GC1, p2P24-GC4, p2P24-GC8, p2P24-GC26, p2P24-GC30, p2P24-GC28-1, and p2P24-28-2 (Supplementary Table S1). These were introduced individually into the *E. coli* DH5α donor strain then transferred into *Pseudomonas* sp. 11K1 by triparental mating (Thoma and Schobert, 2009). In brief, cells from 300 μL of overnight LB donor, helper DH5α (λπ) containing plasmid pRK600, and recipient cultures were washed twice with ddH_2_O and resuspended in 300 μL of ddH_2_O. The three suspensions were pooled (1:1:1) and cells were harvested by centrifugation. Bacterial pellets were resuspended in 100 μL of ddH_2_O and spotted onto an LB plate (2 μL per droplet). After incubation for 6 h at 28°C, bacterial cells were removed from the medium, resuspended in 1 mL of ddH_2_O, and 100 μL aliquots or serial dilutions were plated on ABM plates supplemented with kanamycin.

To further confirm the antimicrobial activities of lipopeptides, we constructed in-frame deletion mutants of gene clusters encoding brasmycin, braspeptin and brasamide, and combinations thereof (double and triple mutants). In-frame deletions were made using a two-step homologous recombination strategy. Briefly, ∼1 kb upstream and downstream fragments of brasmycin, braspeptin, and brasamide gene clusters were PCR-amplified from 11K1 genomic DNA using primers listed in Supplementary Table S2. PCR products were digested with restriction enzymes and cloned into the suicide vector p2P24-Km to generate plasmids p2P24-Δbam, p2P24-Δbap, and p2P24-Δbaa, which were individually introduced into strain 11K1 by triparental mating. Colonies appeared on LB plates containing kanamycin were picked up and inoculated in liquid LB medium without antibiotics. Overnight cultures were then plated on LB plates supplemented with 20% sucrose to generate the mutants Δbam, Δbap, and Δbaa, in which 38,432, 73,251, and 26,622 bp regions of the biosynthesis gene clusters were removed, respectively (Yan et al., 2017). All deletion mutants were screened and confirmed by PCR amplification (Supplementary Figure S1). The same method was used to generate double gene cluster deletion mutants ΔbamΔbap, ΔbamΔbaa, and ΔbapΔbaa, and the triple gene cluster deletion mutant ΔbamΔbapΔbaa (Supplementary Table S1).

### Identification and Analysis of Domains in NRPS Genes

The antiSMASH 4.0 program was used for cluster analysis of the A, T, and C domains of predicted NRPS genes. Sequences from predicted A domains in 11K1 and known A domains from CLPs in other pseudomonads were aligned using ClustalW. Phylogenetic trees were inferred by the neighbor joining method using MEGA5 with 1,000 bootstrap replicates. The peptide moieties of putative CLP signature sequences identified in the A domains were predicted by NRPSpredictor2 as described previously (Röttig et al., 2011).

### Extraction of Lipopeptides

Overnight liquid cultures of 11K1 in LB were spread onto PDA plates (100 μL). After incubation for 5 days at 28°C, agar was sliced into small pieces and extracted with water overnight at 28°C with shaking at 180 rpm in a 500 mL Erlenmeyer flask. The water extract was separated from agar by cheesecloth filtration and subsequently from cell debris by centrifugation (7000 *g*, 10 min) (Michelsen et al., 2015a,b). The supernatant pH was adjusted to pH 2 using 6 M HCl, and the sample was incubated overnight at 4°C before centrifugation (7000 *g*, 10 min). The pellet was collected and dried at 50°C, the crude peptide extract was resuspended in 1 mL of methanol (Biniarz et al., 2016), and analyzed by matrix-assisted laser desorption/ionization time-of-flight (MALDI-TOF) mass spectrometry (MS).

### Matrix-Assisted Laser Desorption/Ionization Time-of-Flight (MALDI-TOF) MS Analysis

MALDI-TOF MS analysis was conducted on a Bruker UltrafleXtreme MALDI-TOF/TOF MS instrument (Ultraflextreme, Bruker, Germany) operated in reflection-positive ion mode with an acceleration voltage of 20 kV. The sample was mixed with matrix in a 1:1 (v/v) ratio. The matrix was α-cyano-4-hydroxycinnamic acid, which was dissolved in 50% acetonitrile containing 0.1% trifluoroacetic acid (TFA) (Monti et al., 2011). The nitrogen laser was set at a threshold adequate for signal generation that minimized fragmentation.

### Swarming Motility and Biofilm Formation

Swarming motility was studied by spotting 5 μL of an overnight cell suspension on KBG medium solidified with 0.6% agar, and evaluating surface swarming motility after incubation at 28°C for 24 h (Olorunleke et al., 2017). Biofilm formation was quantified by incubating 11K1 and its mutants for 3 days at 28°C in glass tubes, and staining with 0.1% crystal violet. Stained biofilms on the inner-surface of glass tubes were subsequently extracted with 95% ethanol and quantified by measuring the absorbance of the solution at 570 nm (Spiers et al., 2003).

## Results

### Genomic Features of *Pseudomonas* sp. 11K1

The 11K1 complete genome consists of a 6,682,832 bp chromosome and one 22,045 bp plasmid, p11K1. A total of 5,785 CDSs were predicted, of which 5,749 CDSs are chromosomal and 36 are encoded on the plasmid. 84 RNA genes including rRNA, tRNA and tmRNA genes were identified. In addition, 81 miscellaneous RNA genes were identified. The general features of the strain 11K1 genome are summarized in Table 1. Based on 16S rDNA sequence analysis, strain 11K1 was identified as a species in the *P. fluorescens* group (Supplementary Figure S2; Hesse et al., 2018).

**Table 1:** Genome statistics for *Pseudomonas* sp. 11K1.

Feature	Chromosome	Plasmid	Total
Size (bp)	6,682,832	22,045	6,704,877
G+C content	60.3	53.2	60.3
Number of genes	5,913	37	5,950
Number of CDSs	5,749	36	5,785
Number of rRNAs	16	0	16
Number of tRNAs	67	0	67
Number of tmRNAs	1	0	1
Number of miscellaneous RNAs	80	1	81

### Automated Searching for Secondary Metabolite Clusters Using the antiSMASH 4.0 Pipeline

The genome of 11K1 was subjected to an automated search using antiSMASH (version 4.0). When the “Extra Features” settings were applied, 10 gene clusters were identified (Table 2), five of which are NRPS-type gene clusters predicted to be involved in the biosynthesis of lipopeptides, pyoverdine, and cupriachelin, four were predicted to synthesize a bacteriocin, a homoserine lactone (Hserlactone), a aryl polyene, and a lantipeptide, and one designated “Other” accounts for biosynthesis of mangotoxin (Table 2). An “Extended” search found an additional 33 gene clusters (Supplementary Table S3), 24 of which had no predicted products or functions. Other clusters showed only low gene similarities (<40%) with known biosynthetic clusters except for gene cluster 39, which shares 80% gene similarities to the alginate BGC (Supplementary Table S3).

**Table 2:** Secondary metabolite and antibiotic gene clusters in *Pseudomonas* sp. 11K1 predicted by antiSMASH 4.0^a^.

Cluster^b^	Type	Most similar known cluster^c^	MIBiG BGC-ID^d^
Cluster 1	Other	Mangotoxin biosynthetic gene cluster (71% of genes show similarity)	BGC0000387_c1
Cluster 4	Aryl polyene	APE Vf biosynthetic gene cluster (40% of genes show similarity)	BGC0000837_c1
Cluster 8	Bacteriocin	–	–
Cluster 14	NRPS	Pyoverdine biosynthetic gene cluster (11% of genes show similarity)	BGC0000413_c1
Cluster 26	NRPS	Syringopeptin biosynthetic gene cluster (100% of genes show similarity)	BGC0000438_c1
Cluster 28	NRPS	Syringomycin biosynthetic gene cluster (100% of genes show similarity)	BGC0000437_c1
Cluster 30	Hserlactone	–	–
Cluster 32	NRPS	Cupriachelin biosynthetic gene cluster (17% of genes show similarity)	BGC0000330_c1
Cluster 35	NRPS	Pyoverdine biosynthetic gene cluster (20% of genes show similarity)	BGC0000413_c1
Cluster 41	Lantipeptide		

*^*a*^Clusters identified by antiSMASH 4.0 using the “Extra Features” settings and the gene clusters highlighted in gray were inactivated by site-directed insertional mutagenesis. ^*b*^Cluster 28 contains two CLP gene clusters. ^*c*^The percentage similarity between genes in predicted clusters and the most similar known cluster. A BLAST *e*-value <1E-05 was used as a significance threshold for genes showing similarity, along with 30% minimal sequence identity, and a shortest BLAST alignment coverage >25% of the sequence. ^*d*^Hyperlinks to the MIBiG repository.*

**FIGURE 1:**
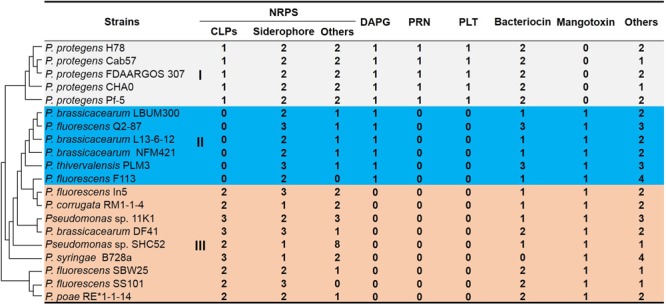
*In silico* analysis and comparison of secondary metabolite production in *Pseudomonas* sp. 11K1 and strains of related species. Clusters accounting for biosynthesis of secondary metabolites were predicted by antiSMASH 4.0 using the “Extra Features” settings. Numbers represent the number of gene clusters within a genome. Abbreviations are as follows: CLPs, cyclic lipopeptides; DAPG, 2,4-diacetylphloroglucinol; PRN, pyrrolnitrin; PLT, pyoluteorin; NRPS, non-ribosomal peptide synthase. *Pseudomonas* strains were divided into groups I (gray), II (blue), and III (orange) based on profiles of secondary metabolites. Group I species produce all four antimicrobial compounds DAPG, PLT, PRN, and CLPs; *Pseudomonas* strains of Group II produce DAPG but not PLT, PRN, or CLPs; *Pseudomonas* strains of group III, including strain 11K1, produce CLPs but not DAPG, PLT, or PRN. The phylogenetic tree of 16S rDNA sequences (left) was constructed using the neighbor-joining method in MEGA version 5.0 after multiple sequence alignment by ClustalW.

We compared the secondary compound profile as predicted for strain 11K1 with 19 other *Pseudomonas* strains, and siderophore production is the only common feature present in all strains (Figure 1). Other antimicrobial compounds (e.g., CLPs, DAPG, PLT, and PRN) are present in conserved genomic locations of some closely related strains (Figure 1). Group III *Pseudomonas* spp. including strain 11K1 all produce CLPs but not DAPG, PLT, or PRN (Figure 1). *Pseudomonas* strains from Group II only produce DAPG but not PLT, PRN, or CLPs (Figure 1). Group I, comprising five *P. protegens* strains, produces all four types of antimicrobial compounds (DAPG, PLT, PRN, and CLPs; Figure 1). These results indicate that different *Pseudomonas* strains, even those belonging to the same species, may produce different secondary compounds. Closely related strains share similar metabolite profiles, and strain 11K1 may produce multiple CLPs with similar chemical structures to the compounds produced by other members of Group III.

### Identification of Gene Clusters Involved in Antibiosis Activity

**FIGURE 2:**
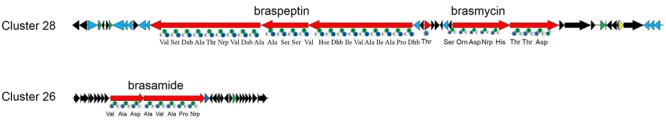
The CLP gene clusters of *Pseudomonas* sp. 11K1. CLPs are synthesized by modular non-ribosomal peptide synthases (NRPSs), and each module contains an adenylation (A), condensation (C), and thiolation (T) domain. Red arrows indicate biosynthetic genes, blue arrows indicate transport-related genes, green arrows indicate regulatory genes, the yellow arrow indicates a predicted transposase, and other genes are indicated by black arrows.

Based on the results of the *in silico* analysis, we performed site-directed insertional mutagenesis of seven gene clusters (Table 2), to find out whether one or more of these clusters contribute to the antimicrobial activity of strain 11K1. Inactivated clusters included NRPS-type clusters 26 and 28. The latter cluster is predicted to produce two CLPs. The two CLPs synthetic gene clusters in cluster 28 are located next to each other, but their synthetic genes transcribe in opposite direction (Figure 2). The growth of *B. dothidea* was not affected by any of the site-directed insertional mutants except mutants 11K1-GC28-1::Km and 11K1-GC28-2::Km. These mutants were both hit in cluster 28. The former mutant displayed a reduced inhibition phenotype whereas the latter completely failed to inhibit *B. dothidea*. These results indicate that the two CLPs encoded by gene cluster 28 are potential antifungal compounds. The first of the CLP gene clusters in cluster 28 was predicted to encode a 22 amino acid lipopeptide, and the second was predicted to encode a 9 amino acid lipopeptide. Gene cluster 28 is similar to the syringomycin/syringopeptin BGC in *P. syringae* pv. *syringae* B301D, and the nunamycin/nunapeptin BGC in *P. fluorescens* In5 (Supplementary Figure S3A). The third CLP, encoded by gene cluster 26, was predicted to encode an 8 amino acid lipopeptide, and this cluster shares similarity with the syringopeptin gene cluster in *P. syringae* B301D and the cichopeptin gene cluster in *P. cichorii* SF1-54 (Scholz-Schroeder et al., 2001; Huang et al., 2015; Supplementary Figure S3B). We designated the predicted 22 amino acid CLP as braspeptin (bap), the 9 amino acid compound as brasmycin (bam), and the 8 amino acid peptide as brasamide (baa; Figure 2). All mutants retained their inhibitory activity against *X. oryzae* RS105, indicating that other compounds, but not CLPs, were responsible for inhibition of this bacterium.

**FIGURE 3:**
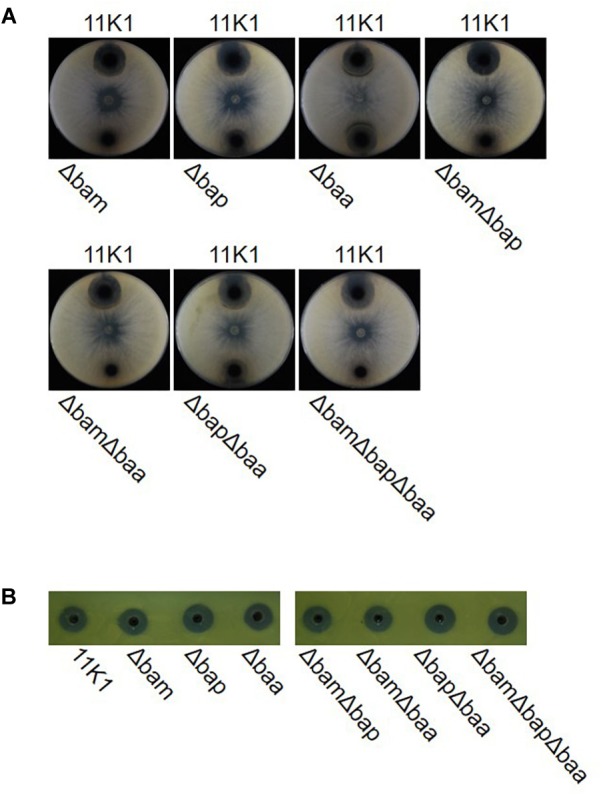
**(A)** Antimicrobial activity of *Pseudomonas* sp. 11K1 and its mutants against *Botryosphaeria dothidea*, and **(B)** *Xanthomonas oryzae* RS105. *B. dothidea* disks (5 mm diameter) were placed in the center of each plate and 5 μL of an overnight culture of wild-type (WT) 11K1 or its mutants were inoculated near the edge of PDA medium plates. For antibacterial assays, 50 μL of sterile fermentation liquor was used to detect bacterial inhibition. Δbam, brasmycin gene cluster deletion mutant; Δbap, braspeptin gene cluster deletion mutant; Δbaa, brasamide gene cluster deletion mutant; ΔbamΔbap, brasmycin and braspeptin double gene cluster deletion mutant; ΔbamΔbaa, brasmycin and brasamide double gene cluster deletion mutant; ΔbapΔbaa, braspeptin and brasamide double gene cluster deletion mutant; ΔbamΔbapΔbaa, brasmycin, braspeptin, and brasamide triple gene cluster deletion mutant.

To further evaluate the role of CLPs in antibiosis activity, we constructed deletion mutants of single gene clusters Δbam, Δbap, and Δbaa, double gene clusters ΔbamΔbap, ΔbamΔbaa, and ΔbapΔbaa, and the triple gene cluster ΔbamΔbapΔbaa. Mutants Δbam, ΔbamΔbap, ΔbamΔbaa, and ΔbamΔbapΔbaa failed to inhibit *B. dothidea* growth, whereas mutant Δbaa retained full antifungal activity comparable with that of the WT 11K1 strain. Mutants Δbap and ΔbapΔbaa displayed a reduced inhibition phenotype compared with the Δbaa and WT strains (Figure 3A). These results confirm the results of the insertional mutation analysis, and indicate that brasmycin is the major antifungal compound active against *B. dothidea*. In addition, all CLP deletion mutants retained antibacterial activity against *X. oryzae* RS105 (Figure 3B), consistent with the above results obtained using insertional mutants, further confirming that CLPs were not the antibacterial compounds active against *X. oryzae* RS105.

### Sequence-Based Structure Prediction of Brasmycin and Braspeptin

**FIGURE 4:**
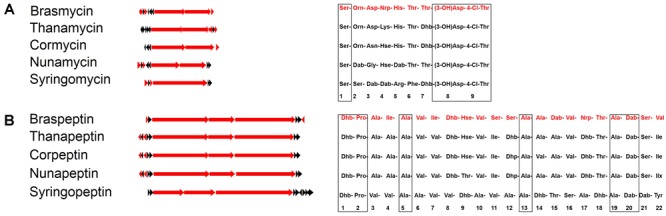
Comparison of brasmycin **(A)** and braspeptin **(B)** gene clusters and the predicted amino acid sequences with those of similar CLPs. Red arrows indicate biosynthesis genes, while other genes are indicated by black arrows. Amino acid sequences of brasmycin and braspeptin (in red) were predicated based on A domain phylogenetic analysis. Cormycin and corpeptin from *P. corrugata* CFBP5454 (ATKI00000000), nunamycin and nunapeptin from *P. fluorescens* In5 (LIRD01000000), thanamycin and thanapeptin from *Pseudomonas* sp. SHC52 (CBLV000000000), syringomycin and syringopeptin from *P. syringae* pv. *syringae* B301D (CP005969). Consensus amino acids are denoted in boxes. Non-standard amino acids are abbreviated as follows: Dab, 2,4-diaminobutyric acid; Dhb, dehydrobutyrine; Dha, dehydroalanine; Orn, ornithine; Hse, homoserine, Nrp, uncertain amino acids.

Bioinformatic analysis revealed that the predicted brasmycin BGC comprises nine modules, each of which consists of an adenylation (A), a thiolation (T), and a condensation (C) domain. Similar to nunamycin and syringomycin BGCs, the last module lacks the A domain, encoded by the first gene (Figure 2; Raaijmakers et al., 2006). The amino acid composition of CLPs could be predicated *in silico* based on A domain selectivity (Challis et al., 2000), and various A domain specificity features have been empirically determined (Röttig et al., 2011). We predicted the amino acid sequence of brasmycin based on a phylogenetic approach in which all A domains were compared with those of functionally characterized lipopeptides. The predicted brasmycin amino acid sequence was Ser-Orn-Asp-Nrp-His-Thr-Thr-Asp-Thr (Figure 4A and Supplementary Figure S4), which is most similar to thanamycin, although the fourth amino acid was uncertain. The predicted brasmycin sequence differs from nunamycin at amino acid 2 (Orn versus Dab), 3 (Asp versus Gly), and 5 (His versus Dab) and from syringomycin at amino acid 2 (Orn versus Ser), 3 (Asp versus Dab), 5 (His versus Arg), 6 (Thr versus Phe), and 7 (Thr versus Dhb) (Figure 4A), suggesting that brasmycin may represent a new member of the syringomycin group (Gross and Loper, 2009). These results are in agreement with a previous report showing that amino acids 2–7 of syringomycin group members are the most variable (Michelsen et al., 2015b).

The predicted amino acid sequence of braspeptin based on A domain phylogenetic analysis suggested that it belonged to the tolaasin group, but with differences from known members (Figure 4B and Supplementary Figure S5). Braspeptin shares conserved sites at amino acid 1 (Dhb), 2 (Pro), 5 (Ala), 13 (Ala), 19 (Ala), and 20 (Dab) with corpeptin, nunapeptin, thanapeptin, and syringopeptin, but differs from corpeptin at amino acid 4 (Ile versus Ala), 7 (Ile versus Val), 11 (Ser versus Ile), 12 (Ser versus Dhp), 15 (Dab versus Ala), and 22 (Val versus Ile); it differs from nunapeptin at amino acid 4 (Ile versus Ala), 7 (Ile versus Ala), 9 (Hse versus Thr), 11 (Ser versus Ile), 12 (Ser versus Dhp), 15 (Dab versus Ala), and 22 (Val versus Ilx); it differs from thanapeptin at amino acid 4 (Ile versus Ala), 7 (Ile versus Val), 11 (Ser versus Ile), 12 (Ser versus Dhb), 15 (Dab versus Ala), and 22 (Val versus Ile); it differs from syringpeptin at amino acid 3 (Ala versus Val), 4 (Ile versus Val), 6 (Val versus Ala), 7 (Ile versus Val), 8 (Dhb versus Val), 9 (Hse versus Dhb), 10 (Val versus Ala), 11 (Ser versus Val), 12 (Ser versus Ala), 14 (Ala versus Dhb), 15 (Dab versus Thr), 16 (Val versus Ser), 18 (Thr versus Dhb), 21 (Ser versus Dab), and 22 (Val versus Tyr). Thus, although amino acid 17 remains uncertain (Figure 4B), braspeptin can be considered a new member of the tolaasin group.

### Identification of CLPs in *Pseudomonas* sp. 11K1 Using MALDI-TOF MS

**FIGURE 5:**
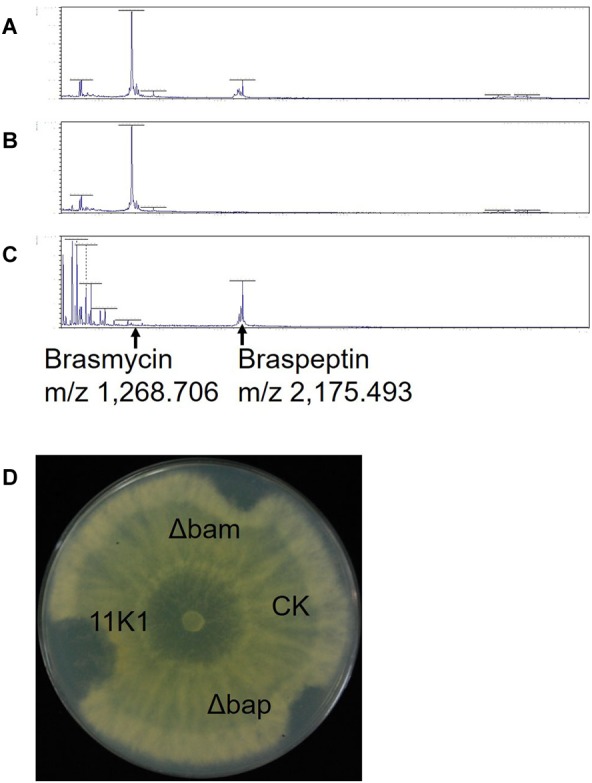
MALDI-TOF MS analysis of CLPs extracted from **(A)** *Pseudomonas* sp. 11K1, **(B)** braspeptin gene cluster mutant Δbap, and **(C)** brasmycin gene cluster mutant Δbam. **(D)** Antifungal analysis of crude CLP extracts. Inhibition of *B. dothidea* mycelial growth by crude peptide extracts derived from *Pseudomonas* sp. 11K1 and its mutants was assessed. Extracts (20 μL) from 11K1, braspeptin gene cluster deletion mutant Δbap, and brasmycin gene cluster deletion mutant Δbam were spotted on agar plates. Methyl alcohol was applied as a control (CK).

Lipopeptides from *Pseudomonas* sp. 11K1 contributing to its antibiosis activity were extracted, and analyzed using a Bruker UltrafleXtreme MALDI-TOF/TOF MS instrument to determine their molecular masses. In WT 11K1, two peaks were obtained at m/z 1,268.706 and 2,175.493 (Figure 5A), whereas only the m/z 2,175.493 peak was detected for the brasmycin gene cluster deletion mutant Δbam (Figure 5B), and only m/z 1,268.706 was detected in braspeptin gene cluster deletion mutant Δbap (Figure 5C). These results showed that the m/z 1,268.706 ion corresponds to the brasmycin lipopeptide, and the m/z 2,175.493 ion corresponds to the braspeptin lipopeptide. Molecular weights differ between brasmycin (m/z 1,268.706) and known syringomycin members nunamycin (m/z 1,138) (Michelsen et al., 2015a), thanamycin (m/z 1,291) (Van Der Voort et al., 2015), syringomycins (m/z 1,225, m/z 1,253) (Monti et al., 2011), and cormycin A (m/z 1274) (Scaloni et al., 2004). The molecular weight of braspeptin (m/z 2,175.493) is different from nunapeptins (m/z 2,023.22, m/z 2,037.24, and m/z 2,075.22) (Michelsen et al., 2015a), thanamycin (m/z 2,120.9) (Van Der Voort et al., 2015), syringopeptins (m/z 2,400, m/z 2,428) (Monti et al., 2011), corpeptins (m/z 2,095, m/z 2,121.2) (Licciardello et al., 2012; Strano et al., 2015), and sclerosis (m/z 2,095.3, m/z 2,123.3, m/z 2,145.3) (Berry et al., 2012). These results further confirm that brasmycin and braspeptin represent new members of the syringomycin and tolaasin lipopeptide groups, respectively.

Although it is currently impossible to accurately predict the fatty acid structures of brasmycin and braspeptin, we made a rough prediction based on molecular weights and predicted oligopeptide chains, in which uncertain amino acids were replaced with the most likely amino acids. The brasmycin might have a 12–15 carbon fatty acid tail (Supplementary Figure S6), and the fatty acid tail of braspeptin might contain 10–12 carbon atoms (Supplementary Figure S7).

Corroborating the results from dual-culture experiments (Figure 3A), crude peptides extracted from *Pseudomonas* sp. 11K1 containing both brasmycin and braspeptin, brasmycin extracted from mutant Δbap, and braspeptin extracted from mutant Δbam all inhibited mycelial growth of *B. dothidea* (Figure 5D).

### Lipopeptides Affect Swarming Motility and Biofilm Formation in *Pseudomonas* sp. 11K1

*Pseudomonas* sp. 11K1 was able to swarm on soft KBG plates containing 0.6% agar. All three single lipopeptide gene cluster mutants Δbam, Δbap, and Δbaa exhibited reduced swarming motility to some degree, indicating that all three lipopeptides contribute to swarming motility (Supplementary Figure S8). However, double mutants ΔbamΔbap, ΔbamΔbaa, and ΔbapΔbaa, and triple mutant ΔbamΔbapΔbaa, did not further impair swarming motility (Supplementary Figure S8), indicating that lipopeptides were not the only surfactants contributing to swarming motility; other unknown surfactants were presumably produced under these conditions.

**FIGURE 6:**
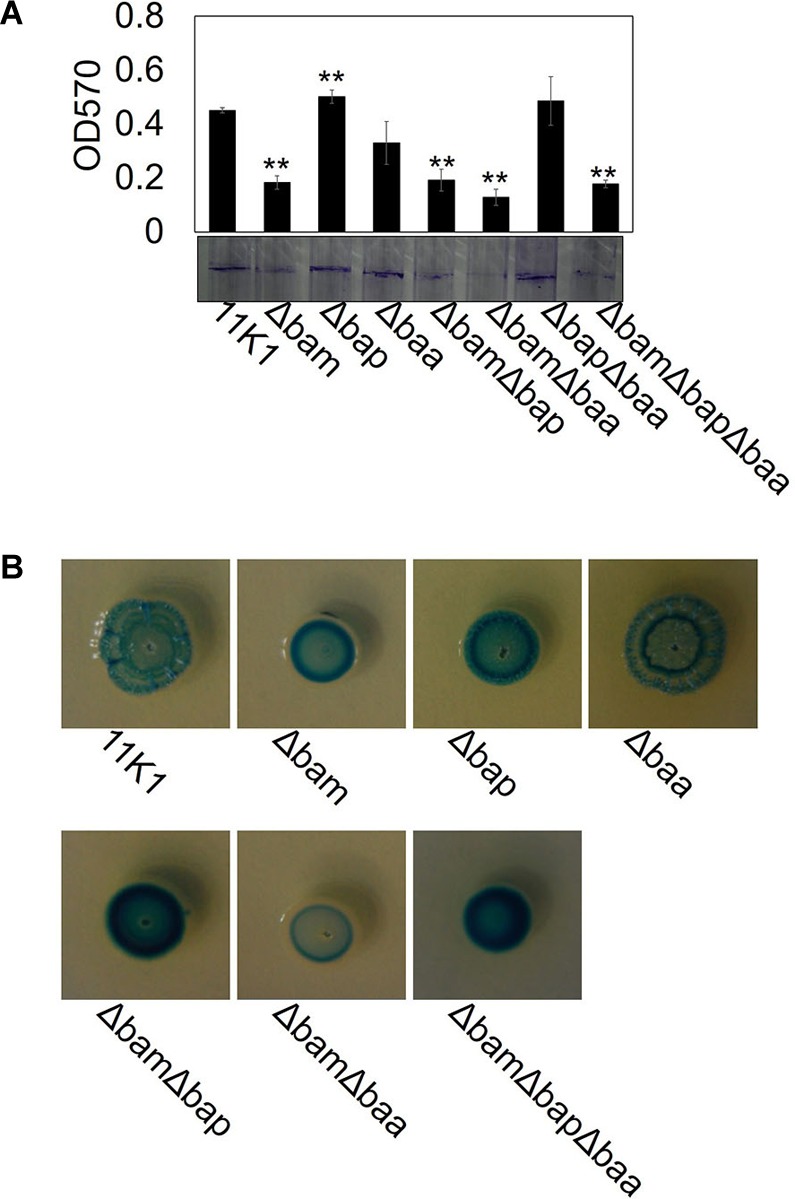
Effects of CLP gene mutations on bacterial biofilm formation **(A)** and colony morphology **(B)**. **(A)** Spectrophotometric quantification of biofilms formed by *Pseudomonas* sp. 11K1 and CLP mutants. Mean values of three replicates are given, and error bars indicate standard error. Visual representation of biofilm formation by *Pseudomonas* sp. 11K1 and CLP mutants on tubes is shown. Biofilms were stained with Crystal Violet. **(B)** Colony morphology detected on PDA plates. A 5 μL sample of overnight culture of WT 11K1 or mutant strains was spot-inoculated in the center of a PDA plate and incubated for 48 h at 28°C. Δbam, brasmycin gene cluster deletion mutant; Δbap, braspeptin gene cluster deletion mutant; Δbaa, brasamide gene cluster deletion mutant; ΔbamΔbap, brasmycin and braspeptin double gene cluster deletion mutant; ΔbamΔbaa, brasmycin and brasamide double gene cluster deletion mutant; ΔbapΔbaa, braspeptin and brasamide double gene cluster deletion mutant; ΔbamΔbapΔbaa, brasmycin, braspeptin, and brasamide triple gene cluster deletion mutant.

Biofilm formation was tested in glass test tubes containing KGB liquid medium. Strain 11K1 formed a biofilm on the inner wall of tubes at the interface between air and liquid. The brasmycin gene cluster mutant Δbam formed markedly less biofilm, the braspeptin gene cluster mutant Δbap produced more biofilm than the WT 11K1 strain, and biofilm formation by mutant Δbaa was comparable to that of 11K1 (Figure 6A). Double and triple gene cluster mutants ΔbamΔbap, ΔbamΔbaa, and ΔbamΔbapΔbaa formed less biofilm, similar to brasmycin mutant Δbam, indicating that brasmycin was the most important lipopeptide involved in biofilm formation.

Mutation of lipopeptide biosynthesis gene clusters caused morphological changes of bacterial colonies on PDA plates. The WT 11K1 strain produced round colonies with a wrinkled surface, whereas all mutants containing brasmycin gene cluster mutations (Δbam, ΔbamΔbap, ΔbamΔbaa, and ΔbamΔbapΔbaa) formed smaller colonies with a smooth surface (Figure 6B). Mutant Δbaa formed typical WT colonies, whereas mutants Δbap and ΔbapΔbaa produced smaller colonies with a less wrinkled surface than WT colonies (Figure 6B).

## Discussion

Genome comparison of twenty *Pseudomonas* strains highlighted the tremendous diversity of gene sequences encoding secondary metabolites (Figure 1), suggesting strains from various environments encounter selective pressure, resulting in the acquisition or loss of gene clusters during evolution. The 20 *Pseudomonas* strains were clustered into three distinct groups according to secondary metabolite profiles. Compared to the vertical inheritance that is the process of genetic material from the parent to the offspring, horizontal genes transfer occurs between different species (Eisen, 2000). Group III is composed of different *Pseudomonas* species including *P. fluorescens, P. brassicacearum* and strain 11K1, all producing CLPs but not DAPG, PLT, or PRN. In contrast, *P. fluorescens* and *P. brassicacearum* in group II do not produce CLPs (Figure 1), indicating the horizontal transfer possibility of CLP biosynthesis gene clusters among *Pseudomonas* species. In addition, we found one transposase gene flanking the brasmycin and braspeptin synthetic gene cluster, which further indicates CLP gene clusters can be horizontally acquired (Figure 2).

The two active CLPs produced by strain 11K1 (brasmycin and braspeptin) belong to the syringomycin and tolaasin groups, respectively. CLPs in the syringomycin group consist of nine amino acids, including unusual amino acids (Figure 4A). All syringomycin group members possess antifungal ability (Sinden et al., 1971; Michelsen et al., 2015b; Trantas et al., 2015; Van Der Voort et al., 2015), and syringomycin is involved in the full virulence of *P. syringae* pv. *syringae* B301D, since syringomycin (*syrB1*) mutant was significantly reduced in virulence compared with its parental strain (Scholz-Schroeder et al., 2001). Compared with the syringomycin group, CLPs in the tolaasin group are much more diverse, both in the composition of the peptide chain, and in antimicrobial activities (Figure 4B). Syringopeptin and corpeptin display antifungal activity, but not antioomycete activity (Lavermicocca et al., 1997; Strano et al., 2015). By contrast, antifungal activity has not been observed for nunapeptin or thanapeptin, but these compounds inhibit oomycete growth (Michelsen et al., 2015b; Van Der Voort et al., 2015). Braspeptin, produced by 11K1, has antifungal activity, but no antioomycete activity (data not shown). Exactly why CLPs with similar composition exhibit different antimicrobial activities remains unclear. A possible explanation is that small structural changes in the peptide or lipid tail may affect their physicochemical properties and interactions with different types of cellular membranes (Kuiper et al., 2004; Raaijmakers et al., 2010).

Biosurfactants such as CLPs, rhamnolipids, and lipopolysaccharides are essential for bacterial swarming motility (Raaijmakers et al., 2006). Under suitable conditions, bacterial cells differentiate into hyperflagellated swarmers that are more motile on wet and viscous surfaces (Harshey, 2003). Biosurfactants produced by bacteria can change the viscosity of surfaces, thereby influencing cell differentiation and motility (McCarter and Silverman, 1990; Allison et al., 1993). Several studies showed that biosurfactants produced by pseudomonads and other bacteria can promote their surface motility. For example, orfamide and massetolide A are required as surfactants for the swarming ability of *Pseudomonas* strains CMR12a and SBW25, respectively, because swarming was completely abolished in CLP biosynthesis mutants (de Bruijn et al., 2007; D’Aes et al., 2014). Similarly, swarming of *P. aeruginosa* 57RP requires alkanoic acids that act as surfactants (Deziel et al., 2003). *Salmonella enterica* SJW1103 lipopolysaccharide biosynthesis gene mutants lose swarming motility (Toguchi et al., 2000). Our present study shows that the single lipopeptide gene cluster mutants Δbam, Δbap, and Δbaa in 11K1 display partly impaired swarming motility, indicating that all three lipopeptides contribute to swarming motility (Supplementary Figure S8). However, double mutants ΔbamΔbap, ΔbamΔbaa, and ΔbapΔbaa, and triple mutant ΔbamΔbapΔbaa, did not further impair swarming motility (Supplementary Figure S8), indicating that other biosurfactants contribute to the full swarming phenotype (Supplementary Table S3).

Cyclic lipopeptides play an important role in biofilm formation, although this can differ between CLP types. For example, a massetolide A-deficient mutant of *P. fluorescens* SBW25 forms less biofilm (de Bruijn et al., 2007), whereas a putisolvin-deficient mutant of *P. putida* PCL1445 displays enhanced biofilm formation (Kuiper et al., 2004). Orfamide has no effect on biofilm formation in *P. fluorescens* Pf-5 (Gross et al., 2007). In strain 11K1, the brasmycin gene cluster mutant Δbam formed markedly less biofilm, whereas the braspeptin gene cluster mutant Δbap produced more biofilm than the WT 11K1 strain (Figure 6A). The mechanisms by which CLPs affect biofilm formation remain unclear, but because CLPs play an important role in cell surface hydrophobicity, CLPs with different structures might differ in hydrophobicity, and their roles in biofilm formation could be entirely different (de Bruijn et al., 2008; Raaijmakers et al., 2010).

A tight correlation between wrinkled colony morphology and increased biofilm formation has been reported for various bacteria including *P. aeruginosa* PA14, *Burkholderia cenocepacia* H111, and *P. fluorescens* SBW25 (Spiers et al., 2003; Friedman and Kolter, 2004; Fazli et al., 2013), but there are exceptions. *B. subtilis* NCIB 3610 produces robust wrinkled colonies, but displays poor biofilm formation on glass surfaces, whereas *B. subtilis* JH642 readily forms biofilms on glass surfaces, but does not produce robust wrinkled colonies (Vlamakis et al., 2013). In the present work, both Δbam and Δbap mutants produce smooth surface colonies, but only Δbam forms less biofilm than the WT 11K1 strain, which produces wrinkled colonies (Figure 6B). Both biofilm formation and wrinkled colony morphology require exopolysaccharides, but the exopolysaccharides involved are not always the same (Fazli et al., 2014). Although acetylation of cellulose is essential for wrinkled colonies, it is not required for biofilm formation on tubes by *P. fluorescens* SBW25 (Spiers et al., 2003). Thus, the molecular relationship between biofilm formation and wrinkled colony morphology remains unclear.

The gene cluster of brasamide shares high similarity with some predicted CLP gene clusters in strains of the *P. fluorescens* group (Supplementary Figure S9), but the potential CLPs they encode have not yet been investigated. The predicted brasamide and its homologs contain eight amino acids with identical sequence, which are distinctly different from two known eight amino acid CLPs bananamide and pseudofactin (Supplementary Figure S9B; Janek et al., 2010; Nguyen et al., 2016). We cannot assign brasamide to any existing group because its chemical structure has not been experimentally resolved, but the distinctly different composition of predicted amino acids suggests that brasamide may belong to a new group of lipopeptides. Except for its contribution to bacterial swarming (Supplementary Figure S6), few information is available about its biological function. However, conserved distribution of brasamide-like CLPs in closely related pseudomonad strains strongly suggest that these CLPs fulfill an ecological function, which will be subject of future study.

## Data Availability

This complete genome project has been deposited at GenBank under the accession numbers CP035088 (chromosome sequence) and CP035089 (plasmid sequence).

## Author Contributions

HZ and L-QZ designed the research. L-QZ supervised the study. Y-PL contributed to the MALDI-TOF MS. HZ performed the other experiments, analyzed the data, and wrote the manuscript together with L-QZ. All authors revised the manuscript and approved the final version for submission.

## Conflict of Interest Statement

The authors declare that the research was conducted in the absence of any commercial or financial relationships that could be construed as a potential conflict of interest.
