# Supplementary material for: In silico and Genetic Analyses of Cyclic Lipopeptide Synthetic Gene Clusters in Pseudomonas sp. 11K1
Source: Front Microbiol. 2019 Mar 19;10:544. doi: 10.3389/fmicb.2019.00544 (PMC6433849; doi:10.3389/fmicb.2019.00544)
Supplement: Supplementary file 1 [file Data_Sheet_1.pdf]

Supplementary Material

In silico and Genetic Analyses of Cyclic Lipopeptide Synthetic Gene Clusters in *Pseudomonas* sp. 11K1

Hui Zhao[1], Yan-Ping Liu[1,2], Li-Qun Zhang[1*]

*Corresponding author, e-mail address: zhanglq@cau.edu.cn

Supplementary Figure

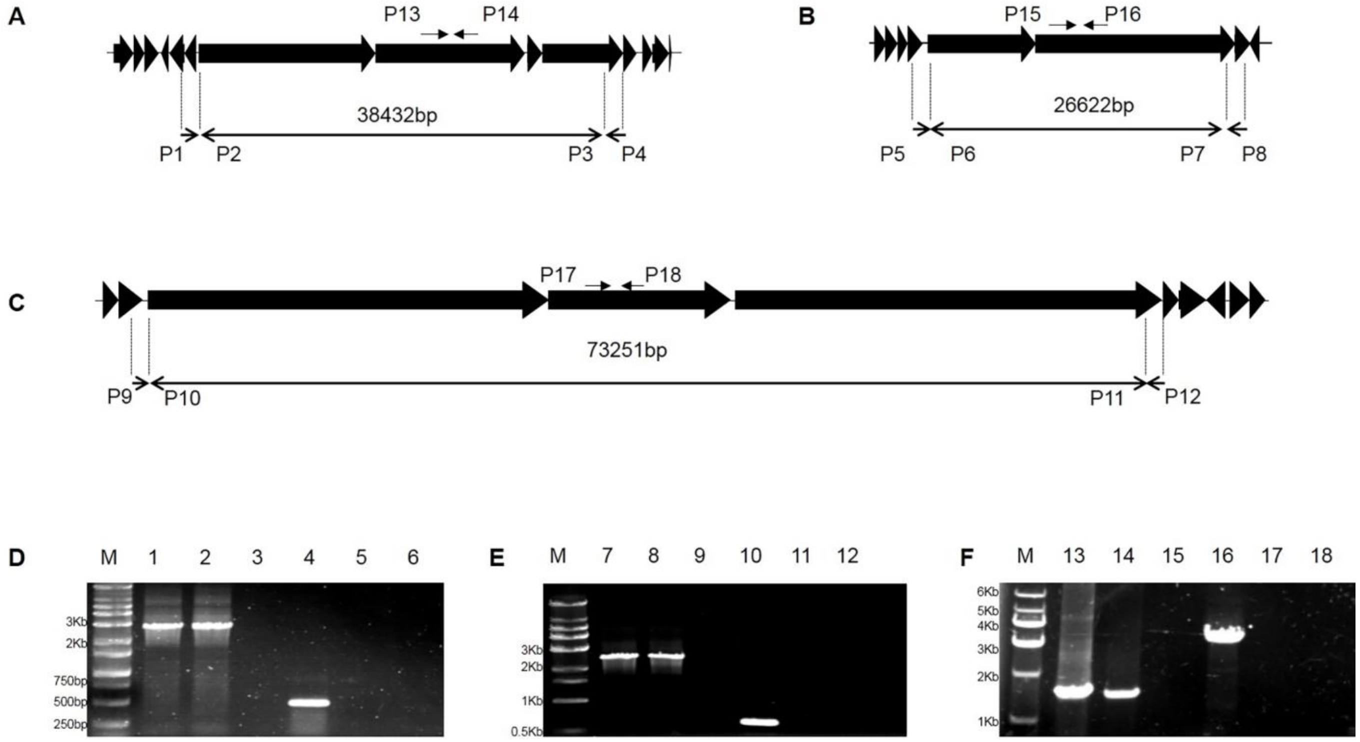

FIGURE S1 | (A) Construction of brasmycin gene cluster deletion mutant Δbam, **(B)** brasamide gene cluster deletion mutant Δbaa, and **(C)** braspeptin gene cluster deletion mutant Δbap. Two rounds of PCR were used to confirm the mutants. **(D)** Agarose gel electrophoresis. Lane 1, plasmid p2P24-Δbam; lane 2, mutant Δbam; lane 3, CK1; lane 4, wild-type (WT) 11K1; lane 5, mutant Δbam; lane 6, CK2. First-round PCR employed primers P1 and P4 for brasmycin mutant detection (lanes 1−3). Second-round PCR employed primers P13 and P14 for brasmycin mutant detection (lanes 4−6). **(E)** Lane 7, plasmid p2P24-Δbaa; lane 8, mutant Δbaa; lane 9, CK1; lane 10, WT 11K1; lane 11, mutant Δbaa; lane 12, CK2. First-round PCR employed primers P5 and P8 for brasamide mutant detection (lanes 7−9). Second-round PCR employed primers P15 and P16 for brasamide mutant detection (lanes 10−12). **(F)** Lane 13, plasmid p2P24-Δbap; lane 14, mutant Δbap; lane 15, CK1; lane 16, WT 11K1; lane 16, mutant Δbap; lane 18, CK2. First-round PCR employed primers P9 and P10 for braspeptin mutant detection (lanes 13−15). Second-round PCR employed primers P17 and P18 for braspeptin mutant detection. M = markers showing the size of DNA standards.
