# Supplementary material for: In silico and Genetic Analyses of Cyclic Lipopeptide Synthetic Gene Clusters in Pseudomonas sp. 11K1
Source: Front Microbiol. 2019 Mar 19;10:544. doi: 10.3389/fmicb.2019.00544 (PMC6433849; doi:10.3389/fmicb.2019.00544)
Supplement: Supplementary file 2 [file Data_Sheet_2.pdf]

In silico and Genetic Analyses of Cyclic Lipopeptide Synthetic Gene Clusters in *Pseudomonas* sp. 11K1

Hui Zhao[1], Yan-Ping Liu[1,2], Li-Qun Zhang[1*]

*Corresponding author, e-mail address: zhanglq@cau.edu.cn

Supplementary Figure

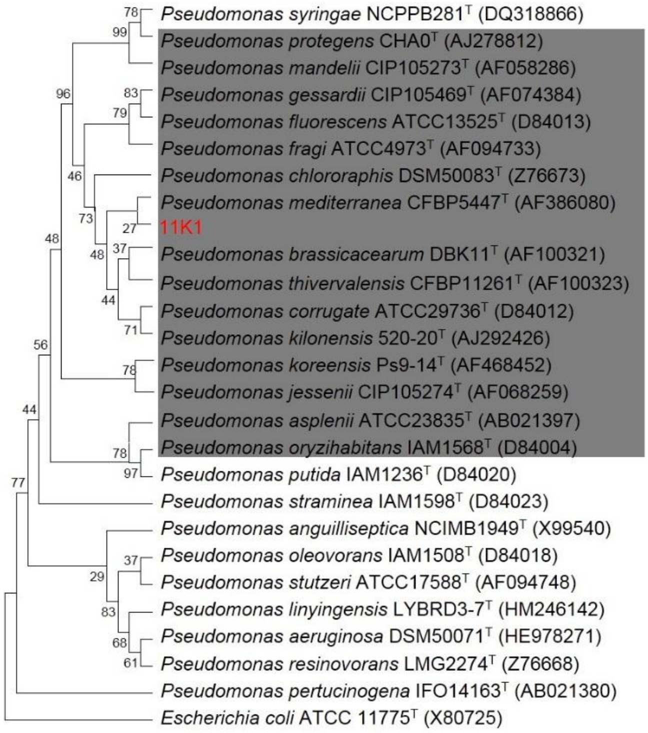

FIGURE S2 | Neighbour-joining phylogeny based on 16S rDNA sequences from *Pseudomonas* species. Phylogenetic analysis was conducted in MEGA5. *Escherichia coli* serves as the out group. The *P. fluorescens* group are highlight in grey (Hesse et al. 2018). T means type strain.

Hesse, C., Schulz, F., Bull, C. T., Elbourne, L. D. H., Yan, Q., Shapiro, N., et al. (2018). Genome‐based evolutionary history of *Pseudomonas* spp. *Environ microbial.* 6(20): 2142-2159 doi: 10.1111/1462-2920.14130
