# Supplementary material for: In silico and Genetic Analyses of Cyclic Lipopeptide Synthetic Gene Clusters in Pseudomonas sp. 11K1
Source: Front Microbiol. 2019 Mar 19;10:544. doi: 10.3389/fmicb.2019.00544 (PMC6433849; doi:10.3389/fmicb.2019.00544)
Supplement: Supplementary file 3 [file Data_Sheet_3.pdf]

Supplementary Material

In silico and Genetic Analyses of Cyclic Lipopeptide Synthetic Gene Clusters in *Pseudomonas* sp. 11K1

Hui Zhao[1], Yan-Ping Liu[1,2], Li-Qun Zhang[1*]

*Corresponding author, e-mail address: zhanglq@cau.edu.cn

Supplementary Figure

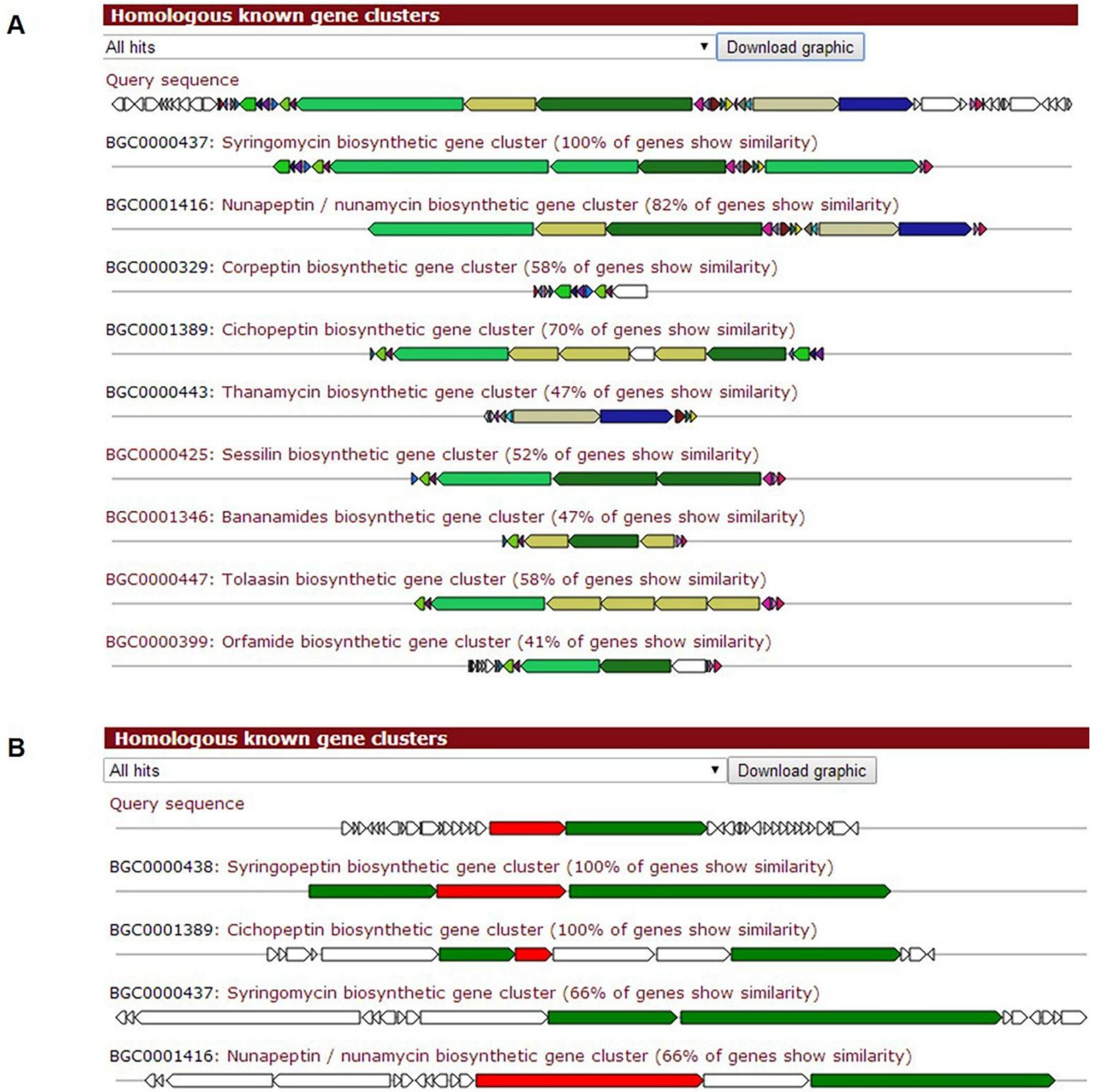

FIGURE S3 | (A) KnownClusterBlast alignment of gene clusters similar to the query gene cluster

(brasmycin and braspeptin). The top nine hits to the brasmycin and braspeptin NRPS gene cluster from *Pseudomonas* sp.11K1 are displayed. **(B)** KnownClusterBlast alignment of gene clusters similar to the query gene cluster (brasamide). The top four hits to the brasamide NRPS gene cluster from *Pseudomonas* sp.11K1 are displayed. Similar genes (BLAST *e*-value <1E-05, 30% minimal sequence identity; shortest BLAST alignment coverage >25% of the sequence) are presented in the same colors. White-colored genes yielded no BLAST hits between gene clusters. The percentage of genes in the query cluster that are present in the hit cluster is included as extra information in brackets. Hyperlinks to the MiBiG repository are included.
