# Supplementary material for: In silico and Genetic Analyses of Cyclic Lipopeptide Synthetic Gene Clusters in Pseudomonas sp. 11K1
Source: Front Microbiol. 2019 Mar 19;10:544. doi: 10.3389/fmicb.2019.00544 (PMC6433849; doi:10.3389/fmicb.2019.00544)
Supplement: Supplementary file 5 [file Data_Sheet_5.pdf]

Supplementary Material

In silico and Genetic Analyses of Cyclic Lipopeptide Synthetic Gene Clusters in *Pseudomonas* sp. 11K1

Hui Zhao[1], Yan-Ping Liu[1,2], Li-Qun Zhang[1*]

*Corresponding author, e-mail address: zhanglq@cau.edu.cn

Supplementary Figure

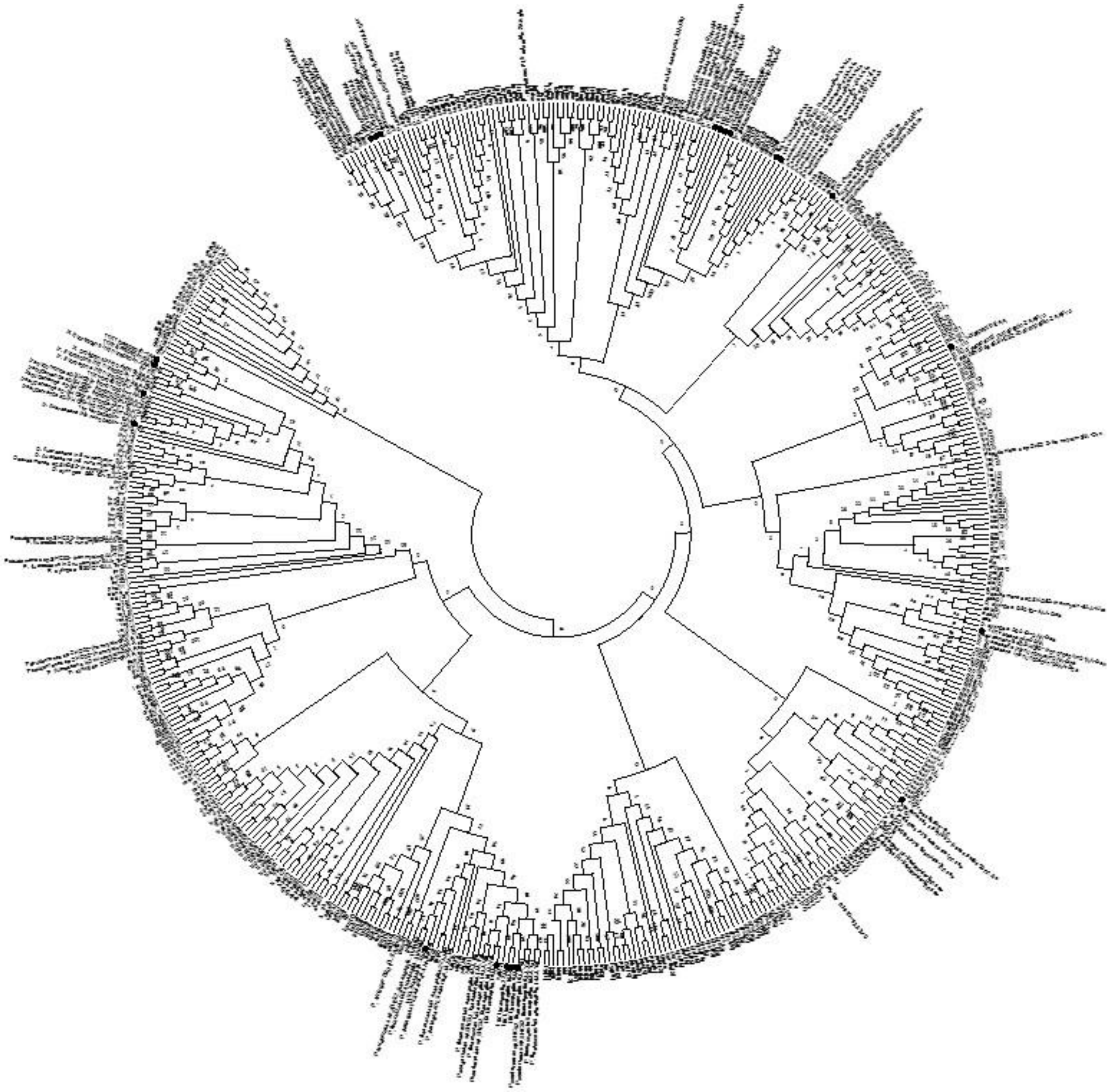

FIGURE S5 | Phylogeny-based substrate specificity prediction of braspeptin A domains. The cladogram is based on the neighbor-joining tree inferred from amino acid sequence alignment of A domains using NRPSpredictor2 (Röttig et al., 2011). Functionally characterized *Pseudomonas* CLPs include nunamycin and nunapeptin from *P. fluorescens* In5, thanamycin and thanapeptin from *Pseudomonas* sp. SHC52, orfamide from *P. protegens* Pf-5, and syringomycin from *P. syringae* pv. *syringae* B301D. Braspeptin A domains are highlighted in black.
