# Supplementary material for: In silico and Genetic Analyses of Cyclic Lipopeptide Synthetic Gene Clusters in Pseudomonas sp. 11K1
Source: Front Microbiol. 2019 Mar 19;10:544. doi: 10.3389/fmicb.2019.00544 (PMC6433849; doi:10.3389/fmicb.2019.00544)
Supplement: Supplementary file 6 [file Data_Sheet_6.pdf]

*Supplementary Material*

# *In silico* and Genetic Analyses of Cyclic Lipopeptide Synthetic Gene Clusters in *Pseudomonas* sp. 11K1

Hui Zhao[1], Yan-Ping Liu[1,2], Li-Qun Zhang[1*]

*Corresponding author, e-mail address: zhanglq@cau.edu.cn

**Supplementary Figure**

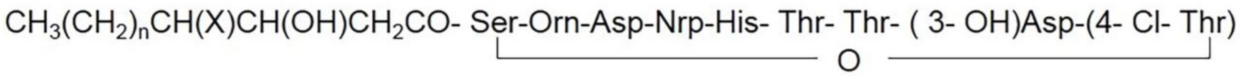


| X | Nrp | n | N (n+5) |
|---|---|---|---|
| OH | Lys | 7.21 | 12.21 |
| H | Lys | 8.36 | 13.36 |
| OH | Hse | 9.15 | 14.15 |
| H | Hse | 10.29 | 15.29 |

**FIGURE S6 |** The predicted structure of brasmycin. Calculation of n was based on estimated amino acid structures and the MALDI-TOF molecular weight of the brasmycin, and the characteristics of syringomycin group CLPs. X means hydroxy or hydrogen atom. N means carbon atom number of fatty acid tail. The uncertain amino acid (Nrp) was replaced with the most likely amino acids (Lys and Hse). Non-standard amino acids are abbreviated as follows: Orn, ornithine; Hse, homoserine.
