# Supplementary material for: In silico and Genetic Analyses of Cyclic Lipopeptide Synthetic Gene Clusters in Pseudomonas sp. 11K1
Source: Front Microbiol. 2019 Mar 19;10:544. doi: 10.3389/fmicb.2019.00544 (PMC6433849; doi:10.3389/fmicb.2019.00544)
Supplement: Supplementary file 7 [file Data_Sheet_7.pdf]

Supplementary Material

In silico and Genetic Analyses of Cyclic Lipopeptide Synthetic Gene Clusters in *Pseudomonas* sp. 11K1

Hui Zhao[1], Yan-Ping Liu[1,2], Li-Qun Zhang[1*]

*Corresponding author, e-mail address: zhanglq@cau.edu.cn

Supplementary Figure

$CH_3(CH_2)_nCH(OH)CH_2CO$-Dhb-Pro-Ala-Ile-Ala-Val-Ile-Dhb-Hse-Val-Ser-Ser-Ala-Ala-Dab-Val-Nrp-Thr-Ala-Dab-Ser-Val (Thr–O–Val ring closure)

Nrp	n	N (n+4)
Dhb	7.14	11.14
Thr	5.85	9.85
Ala	8.00	12.00

FIGURE S7 | The predicted structure of braspeptin. Calculation of n was based on estimated amino acid structures and MALDI-TOF molecular weight of braspeptin, and the characteristics of tolaasin group CLPs. N means carbon atom number of fatty acid tail. The uncertain amino acid (Nrp) was replaced with the most likely amino acids (Dhb, Thr and Ala). Non-standard amino acids are abbreviated as follows: Dab, 2,4-diaminobutyric acid; Dhb, dehydrobutyrine; Hse, homoserine.
