# Supplementary material for: In silico and Genetic Analyses of Cyclic Lipopeptide Synthetic Gene Clusters in Pseudomonas sp. 11K1
Source: Front Microbiol. 2019 Mar 19;10:544. doi: 10.3389/fmicb.2019.00544 (PMC6433849; doi:10.3389/fmicb.2019.00544)
Supplement: Supplementary file 8 [file Data_Sheet_8.pdf]

Supplementary Material

In silico and Genetic Analyses of Cyclic Lipopeptide Synthetic Gene Clusters in *Pseudomonas* sp. 11K1

Hui Zhao[1], Yan-Ping Liu[1,2], Li-Qun Zhang[1*]

*Corresponding author, e-mail address: zhanglq@cau.edu.cn

Supplementary Figure

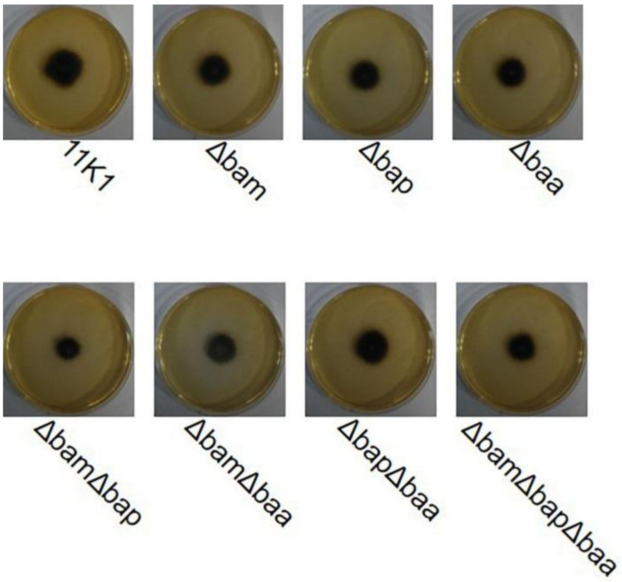

FIGURE S8 | Swarming motility of WT 11K1 and its mutants on soft (0.6% w/v) KBG agar plates. Samples (5 μL) of overnight cultures were spot-inoculated in the center of a soft agar plate and incubated for 24 h at 28°C. Δbam, brasmycin gene cluster deletion mutant; Δbap, braspeptin gene cluster deletion mutant; Δbaa, brasamide gene cluster deletion mutant; Δbam Δbap, brasmycin and braspeptin double gene cluster deletion mutant; Δbam Δbaa, brasmycin and brasamide double gene cluster deletion mutant; Δbap Δbaa, braspeptin and brasamide double gene cluster deletion mutant; Δbam Δbap Δbaa, brasmycin, braspeptin, and brasamide triple gene cluster deletion mutant.
