# Supplementary material for: In silico and Genetic Analyses of Cyclic Lipopeptide Synthetic Gene Clusters in Pseudomonas sp. 11K1
Source: Front Microbiol. 2019 Mar 19;10:544. doi: 10.3389/fmicb.2019.00544 (PMC6433849; doi:10.3389/fmicb.2019.00544)
Supplement: Supplementary file 9 [file Data_Sheet_9.pdf]

Supplementary Material

In silico and Genetic Analyses of Cyclic Lipopeptide Synthetic Gene Clusters in *Pseudomonas* sp. 11K1

Hui Zhao[1], Yan-Ping Liu[1,2], Li-Qun Zhang[1*]

*Corresponding author, e-mail address: zhanglq@cau.edu.cn

Supplementary Figure

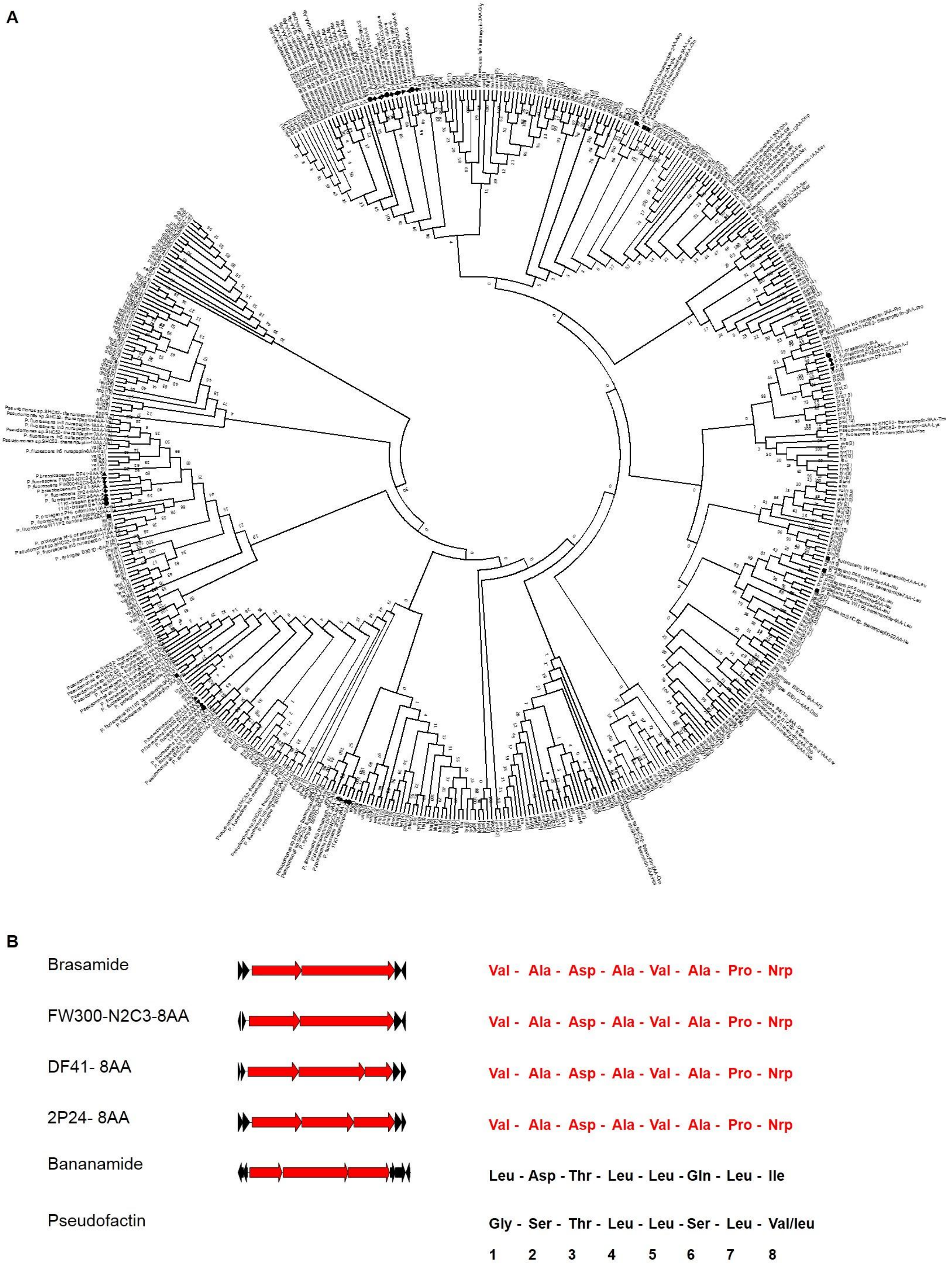

FIGURE S9| (A) Phylogeny-based substrate specificity prediction of brasamide A domains. The cladogram is based on the neighbor-joining tree inferred from amino acid sequence alignment of A domains using NRPSpredictor2 (Röttig et al., 2011). Functionally characterized *Pseudomonas* CLPs include nunamycin

and nunapeptin from *P. fluorescens* In5, thanamycin and thanapeptin from *Pseudomonas* sp. SHC52, orfamide from *P. protegens* Pf-5, and syringomycin from *P. syringae* pv. *syringae* B301D. **(B)** Comparison of the gene cluster and predicted amino acid sequence of brasamide with other predicted and known 8 amino acid CLPs. Bananamide from *P. fluorescens* BW11P2 (KX437753), pseudofactin from *P. fluorescens* BD5, FW300-N2C3-8AA from *P. fluorescens* FW300-N2C3 (CP012831), DF41-8AA from *P. brassicacearum* DF41 (CP007410), 2P24-8AA from *P. fluorescens* 2P24 (CP025542). The predicted amino acids are highlighted in red, and the chemically characterized sequences are in black. The genome sequence of *P. fluorescens* BD5 is not available.
