# Supplementary material for: In silico and Genetic Analyses of Cyclic Lipopeptide Synthetic Gene Clusters in Pseudomonas sp. 11K1
Source: Front Microbiol. 2019 Mar 19;10:544. doi: 10.3389/fmicb.2019.00544 (PMC6433849; doi:10.3389/fmicb.2019.00544)
Supplement: Supplementary file 10 [file Data_Sheet_10.pdf]

Supplementary Material

In silico and Genetic Analyses of Cyclic Lipopeptide Synthetic Gene Clusters in *Pseudomonas* sp. 11K1

Hui Zhao[1], Yan-Ping Liu[1,2], Li-Qun Zhang[1*]

*Corresponding author, e-mail address: zhanglq@cau.edu.cn

Supplementary Table

TABLE S1 | Strains and plasmids used in this study

Strain or Plasmid	Description	Reference or source
Strains		
Escherichia coli		
DH5α	φ80 *lacZ*ΔM15Δ(*lacZYA-argF*)*U169 hsdR17 recA1 endA1 thi-1*	Laboratory stock
DH5α (λ-π)	φ80 *lacZ*ΔM15Δ(*lacZYA-argF*)*U169 hsdR17 recA1 endA1 thi-1 λ-π*	Laboratory stock
Helper	DH5α(λ-π) strain containing pRK600	Laboratory stock
Pseudomonas sp.		This study
11K1	Wild-type, Ap^r	This study
11K1-Δbam	Brasmycin gene cluster in-frame deletion in strain 11K1; Ap^r	This study
11K1-Δbap	Braspeptide gene cluster in-frame deletion in strain 11K1; Ap^r	This study
11K1-Δbaa	Brasamide gene cluster in-frame deletion in strain 11K1; Ap^r	This study
11K1-ΔbamΔbap	Brasmycin and Braspeptide double gene clusters in-frame deletion in strain 11K1; Ap^r	This study
11K1-ΔbamΔbaa	Brasmycin and Brasamide double gene clusters in-frame deletion in strain 11K1; Ap^r	This study
11K1-ΔbapΔbaa	Braspeptin and Brasamide double gene clusters in-frame deletion in strain 11K1; Ap^r	This study
11K1-ΔbamΔbap Δbaa	Brasmycin, braspeptide and brasamide triple gene clusters in-frame deletion in strain 11K1; Ap^r	This study
11K1-ΔGC1::Km	Site-directed insertional mutant on gene cluster 1; Ap^r, Km^r	This study
11K1-ΔGC4::Km	Site-directed insertional mutant on gene cluster 4; Ap^r, Km^r	This study
11K1-ΔGC8::Km	Site-directed insertional mutant on gene cluster 8; Ap^r, Km^r	This study
11K1-ΔGC26::Km	Site-directed insertional mutant on gene cluster 26; Ap^r, Km^r	This study
11K1-ΔGC28-1::Km	Site-directed insertional mutant on gene cluster 28-1; Ap^r, Km^r	This study
11K1-ΔGC28-2::Km	Site-directed insertional mutant on gene cluster 28-2; Ap^r, Km^r	This study
11K1-ΔGC30::Km	Site-directed insertional mutant on gene cluster 30; Ap^r, Km^r	This study

TABLE S1| Continued		
Strain or Plasmid	Description	Reference or source
Botryosphaeria dothidea	Fungal pathogen causing grape canker	Laboratory stock
Xanthomonas oryzae RS105	Bacterial pathogen causing bacterial blight of rice	Laboratory stock
Plasmids		
pRK600	ColE1 oriV; RP4; tra +; RP4 oriT; helper plasmid in triparental matings, Cm^r	Finan et al., 1986
p2P24	Suicide vector for generation of gene in-frame deletions, *sacB*, Km^r	Yan et al., 2017
p2P24-Δbam	Suicide plasmid p2P24 containing deletion structure of brasmycin biosynthesis gene cluster, Km^r	This study
p2P24-Δbap	Suicide plasmid p2P24 containing deletion structure of braspeptin biosynthesis gene cluster, Km^r	This study
p2P24-Δbaa	Suicide plasmid p2P24 containing deletion structure of brasamide biosynthesis gene cluster, Km^r	This study
p2P24-GC1	p2P24 bearing an 862 bp fragment of gene cluster 1, which was amplificated using primers GC1-*Eco*RI-20475/GC1-*Xba*I-21336 and inserted in the *Eco*RI-*Xba*I cloning sites	This study
p2P24-GC4	p2P24 bearing a 559 bp fragment of gene cluster 4, which was amplificated using primers GC4-*Eco*RI-20881/GC4-*Xba*I-21440 and inserted in the *EcoR*I-*Xba*I cloning sites	This study
p2P24-GC8	p2P24 bearing a 555 bp fragment of gene cluster 8, which was amplificated using primers GC8-*Kpn*I-5304/GC8-*Xba*I-5858 and inserted in the *Kpn*I-*Xba*I cloning sites	This study
p2P24-GC26	p2P24 bearing a 587 bp fragment of gene cluster 26, which was amplificated using primers GC26-*Kpn*I-46714/GC26-*Xba*I-47300 and inserted in the *Kpn*I-*Xba*I cloning site	This study
p2P24-GC28-1	p2P24 bearing a 678 bp fragment of gene cluster 28-1, which was amplificated using primers GC28-1-*Xba*I-23341/GC28-1-*Eco*RI-24018 and inserted in the *Xba*I-*Eco*RI cloning site	This study
p2P24-GC28-2	p2P24 bearing a 494 bp fragment of gene cluster 28-2, which was amplificated using primers GC28-2-*Eco*RI-126904/GC28-2-*Xba*I-127398 and inserted in the *Eco*RI-*Xba*I cloning site	This study
p2P24-GC30	p2P24 bearing a 574 bp fragment of gene cluster 30, which was amplificated using primers GC-30-*Xba*I-2711/GC-30-*Eco*RI-3284 and inserted in the *Xba*I-*Eco*RI cloning site	This study

Ap^r, Cm^r, and Km^r indicate resistance to ampicillin, chloramphenicol and kanamycin, respectively.

TABLE S2 | Primers used in this study

Primer	Sequence[a]	Size	Reference or source
GC1-*Eco*RI-20475	ATgaattCACTGCCTCAACCGAC	862 bp	This study
GC1-*Xba*I-21336	GAtctagaCGAACGGCTCACAGAC		
GC4-*Eco*RI-20881	ATgaattcTTGATCTCGTCGGTGC	559 bp	This study
GC4-*Xba*I-21440	ATtctagaCAAGCGCAAGATCACCG		
GC8-*Kpn*I-5304	ATggtaccGATCGCATCTGCCTG	555 bp	This study
GC8-*Xba*I-5858	ATtctagaGCCGGTGCGACGAATAAC		
GC26-*Kpn*I46714	ATggtaccTGCCTGTTCATGATCC	587 bp	This study
GC26-*Xba*I-47300	ATtctagaAACACCACCACTGAGCC		
GC28-1-*Xba*I-23341	ATtctagaGGTAGCCGGAATGTTCG	678 bp	This study
GC28-1-*Eco*RI-24018	ATgaattcTTGTTCCTGGTGCATG		
GC28-2-*Eco*RI-126904	ATgaattcGTTCCATCAGCTCAGC	494 bp	This study
GC28-2-*Xba*I-127398	ATtctaaCGCGGATCTTCACCTG		
GC30-*Xba*I-2711	GCtctagaCCTTGCCATCATTCTCG	574 bp	This study
GC30-*Eco*RI-3284	ATgaattcGGGAATGGAGAGCAAC		
P1	ATTaagcttAACAGATAGGTGCGCAAC	1350 bp	This study
P2	ATtctagaATGTTGATCTCACCCCTG		
P3	ATtctagaATGCGCGAACCTCATATC	1339 bp	This study
P4	ATggtacCAAGGCTTGAACGACAG		
P5	ATgaattcCGATGAGAACCTGCC	1225 bp	This study
P6	ATggtaccTGTAAGCCGCACTAACG		
P7	ATggtaccGCCGTCGGTTTGATG	1148 bp	This study
P8	ATTaagcttTCAATCGCTGTGGTGG		
P9	ATTaagcttGTAACGATCATAGGTGGC	612 bp	This study
P10	TAtctagaCAAGCATATCCCTGACCTC		
P11	TAtctagaGCCGATGTGCAGGTATCC	846 bp	This study
P12	ATggtaccATCAAGATCGAAGGGC		
P13	GTTCCATCAGCTCAGC	494 bp	This study
P14	CGCGGATCTTCACCTG		
P15	CCTTGCCATCATTCTCG	574 bp	This study
P16	AGGGAATGGAGAGCAAC		
P17	GTGCCCAACGTACAG	3193 bp	This study
P18	CCTCCTGGCTCAATG		

TABLE S3 | Gene clusters potentially involved in the synthesis of secondary metabolites and antibiotics by *Pseudomonas* sp.11K1, identified using the antiSMASH4.0[a].

Cluster	Type	Most similar known cluster[b]	MIBiG BGC-ID[c]
Cluster 1	Other	Mangotoxin biosynthetic gene cluster (71% of genes show similarity)	BGC0000387_c1
Cluster 2	putative	Pyoverdine biosynthetic gene cluster (1% of genes show similarity)	BGC0000413_c1
Cluster 3	putative	O-antigen biosynthetic gene cluster (20% of genes show similarity)	BGC0000785_c1
Cluster 4	Arylpolyene	APE Vf biosynthetic gene cluster (40% of genes show similarity)	BGC0000837_c1
Cluster 5	saccharide	Lipopolysaccharide biosynthetic gene cluster (36% of genes show similarity)	BGC0000776_c1
Cluster 6	putative	-	-
Cluster 7	putative	-	-
Cluster 8	Bacteriocin	-	-
Cluster 9	putative	-	-
Cluster 10	fatty_acid	-	-
Cluster 11	fatty_acid	-	-
Cluster 12	putative	Lipopolysaccharide biosynthetic gene cluster (10% of genes show similarity)	BGC0000775_c1
Cluster 13	putative	Lipopolysaccharide biosynthetic gene cluster (13% of genes show similarity)	BGC0000775_c1
Cluster 14	NRPS	Pyoverdine biosynthetic gene cluster (11% of genes show similarity)	BGC0000413_c1
Cluster 15	putative	-	-
Cluster 16	putative	-	-
Cluster 17	putative	-	-
Cluster 18	putative	-	-
Cluster 19	putative	-	-
Cluster 20	putative	-	-
Cluster 21	putative	-	-
Cluster 22	putative	-	-
Cluster 23	putative	-	-
Cluster 24	putative	-	-
Cluster 25	putative	-	-
Cluster 26	NRPS	Syringopeptin biosynthetic gene cluster (100% of genes show similarity)	BGC0000438_c1
Cluster 27	fatty_acid	-	-
Cluster 28	NRPS	Syringomycin biosynthetic gene cluster (100% of genes show similarity)	BGC0000437_c1
Cluster 29	putative	-	-
Cluster 30	Hserlactone	-	-

TABLE S3|Continued			
Cluster	Type	Most similar known cluster[b]	MIBiG BGC-ID[c]
Cluster 31	putative	-	-
Cluster 32	NRPS	Cupriachelin biosynthetic gene cluster (17% of genes show similarity)	BGC0000330_c1
Cluster 33	putative	Polysaccharide B biosynthetic gene cluster (6% of genes show similarity)	BGC0001411_c1
Cluster 34	putative	-	-
Cluster 35	NRPS	Pyoverdine biosynthetic gene cluster (20% of genes show similarity)	BGC0000413_c1
Cluster 36	fatty_acid	Svaricin biosynthetic gene cluster (6% of genes show similarity)	BGC0001382_c1
Cluster 37	fatty_acid	-	-
Cluster 38	putative	-	-
Cluster 39	putative	Alginate biosynthetic gene cluster (80% of genes show similarity)	BGC0000725_c1
Cluster 40	saccharide	Burkholderic acid biosynthetic gene cluster (6% of genes show similarity)	BGC0001120_c1
Cluster 41	Lantipeptide		
Cluster 42	putative	-	-
Cluster 43	putative	-	-

a Clusters identified using the ‘Extra Features’ settings of antiSMASH 4.0 are shown in bold and highlighted gray. The remaining putative clusters are the result of an extended antiSMASH 4.0 search, involving the implementation of the ClusterFinder and Use ClusterFinder algorithm for BGC border prediction analysis options. b The percentage sequence similarity of genes in predicted clusters that are present in the most similar known cluster. The significance thresholds of genes showing similarity are BLAST E-value <1E-05, 30% minimal sequence identity, and shortest BLAST alignment coverage >25% of the sequence. c Hyperlinks to the MiBIG repository are included.
